# Precocious eczema and unexpected anaphylaxis to seafood

**DOI:** 10.1186/2045-7022-1-S1-P7

**Published:** 2011-08-12

**Authors:** Guiseppe Menna, Nicola Antonio Romeo

**Affiliations:** 1 ISS -State Hospital - San Marino Republic (RSM), Pediatrics, Borgo Maggiore, San Marino

## Background

Assessment of allergic food reactions may be complicated by cross-reactivity among certain food families and seemingly unrelated allergens. Analysis can identify protein sequence and allergenic properties. Fish and its derived play an important role in nutrition, they may also be a potent food allergen. Gad-c1, Parvalbumin, the major codfish allergen, is considered as a panallergen as in seafood the Tropomyosin (muscle-derived protein) have been recently demonstrated in invertebrates such as cockroaches, mites, shrimp. The clinical symptoms related to IgE-mediated fish allergy are frequently urticaria, angioedema, mild oral symptoms, worsening atopic dermatitis, respiratory symptoms (rinitis, asthma),gastrointestinal (nausea,vomiting). Anaphylaxis may occur.

## Objective

In view of a possible cross-reactivity between food allergens and related allergens from environmental sources.

## Methods

A 4 yrs children, family atopic risk, severe atopic eczema in the first year and anaphylaxis to fish and seafood during first introduction at 4 years. Positive skin test to egg, RAST ovoalbumin 1.13 KUI/L, RAST at age two Grasses 23.2 KUI/L. Anaphylaxis ( Sampson,2003- 2nd) to codfish at first injestion, to shrimp after 3 months.

## Results

Positive skin to house dust mite (HDM) and cockroach mix were reported. At control, positive RASTto grasses, mites, and recombinant Pen1, Gad 1.

## Conclusions

some subjects allergic to HDM or cockroach show substantial IgE antibody reactivity to the major shrimp allergen Pen a 1 (tropomyosin). Based on inhibition with cockroach and dust mite extracts, this reactivity appears to be due to cross-reacting tropomyosins. This patient showed the literature evolution in cross reactivity: precocious sentitization to egg, correlation to mite and linkage to mite and seafood. And asthma? Follow up of course.
